# New Methodology for Evaluating Strength Degradation from Temperature Increase in Concrete Hydration under Adiabatic Conditions

**DOI:** 10.3390/ma17194830

**Published:** 2024-09-30

**Authors:** Adelino V. Lopes, Sergio M. R. Lopes

**Affiliations:** 1 INESC, Department of Civil Engineering, University of Coimbra, 3030-788 Coimbra, Portugal; avlopes@dec.uc.pt; 2 CEMMPRE, ARISE, Department of Civil Engineering, University of Coimbra, 3030-788 Coimbra, Portugal

**Keywords:** cementitious materials, hydration process, hydration heat, adiabatic conditions, concrete strength, mortar strength, portland cement

## Abstract

Cement-based construction materials, commonly known as “cement concrete”, result from the hydration reaction of cement, which releases heat. Numerous studies have examined the heat of cement hydration and other thermal properties of these materials. However, a significant gap in the literature is the assessment of the impact of the hydration temperature on the material’s strength, particularly compressive strength. This work presents an experimental methodology that consistently estimates the temperature evolution of a mixture used to manufacture concrete or mortar during the first hours of Portland cement hydration. The methodology aims to ensure results that correspond to an infinite medium (adiabatic conditions), where there are no heat losses to the surroundings. Results obtained under adiabatic conditions (simulating an infinite medium) indicate that a ready-made mortar (Portland cement: sand: water; 1:2.5:0.5) can reach temperatures of approximately 100 °C after 48 h of hydration. Under these conditions, compressive strength decreases by up to 20%.

## 1. Introduction

Cementitious concrete is a widely used material, but its application is conditioned by certain characteristics, particularly the heat generated during the hydration reactions of cement. When water is added to Portland cement, the hydration process begins, involving a series of chemical reactions between the water and cement, forming strong bonds that give concrete its strength. In civil engineering, it is well known that cement hydration is an exothermic process, meaning it releases heat. If this heat is not properly managed, it can raise the temperature of the concrete mixture, especially in large structures such as dams and bridges, where the heat cannot dissipate quickly. This temperature rise can cause thermal stresses within the structure and between different concrete castings, potentially leading to cracks and reducing the overall strength and durability of the concrete [1]. Problems such as alkali-silica reaction (ASR) and delayed ettringite formation (DEF)-induced swelling are likely to occur in massive structural members [2].

To control temperature increases and prevent potential structural issues, various methods exist, such as using low-heat or modified Portland cement, cooling the concrete ingredients before mixing, employing temperature-rise inhibitors, or post-cooling the concrete after it has been poured [3,4,5,6,7,8,9]. However, before resorting to these control methods, it is crucial to first evaluate the temperature increase and its impact on the material’s mechanical characteristics.

### 1.1. Hydration of Concrete

Several authors [10,11,12,13,14,15] have studied the problem of temperature gradients in large volumes of concrete. The corresponding dissipation into the surrounding environment causes the temperature inside the concrete to be higher than at its surfaces. These gradients generate tensile stresses in the surface concrete, which can lead to cracking. The non-uniform stress variation in concrete arises due to several factors, including the initial temperatures of the materials used, the type and amount of cement, the ambient temperature, the thermal properties of the concrete, the unequal boundary conditions, and the volume geometry. In large masses of concrete, the problem is exacerbated due to the material’s low conductivity, which retains a significant portion of the released heat and dissipates it slowly.

The maximum temperature attained in a concrete element is based on prior knowledge of the heat of hydration of the cement used. Three main methods can be used to determine this: the solution method (EN 196-8:2010 [16]), the Lagavant method (semi-adiabatic calorimetry covered by EN 196-9:2010 [17]), and the isothermal conduction calorimetry method described in EN 196-11:2020 [18], which is now becoming widely used. Custodio et al. [19] studied the heat of hydration of various cement samples typically used in massive structures. Their laboratory tests, which included 25 cement samples from 13 different cements, revealed that the isothermal conduction calorimetry method tends to give slightly lower heat of hydration values compared to the EN 196-11 results at 168 h. They noted a discrepancy of −11% to −18% compared to EN 196-9 results at 120 h and −6% to −15% compared to EN 196-9 results at 41 h. The best correlation between the two test methods was observed when comparing values at 41 h from the semi-adiabatic calorimetry method and at 168 h from the isothermal conduction calorimetry method.

Baran and Pichniarczyk [20] also evaluated the heat of hydration of numerous cement samples from several manufacturing plants in Poland, obtaining values in the range of 287–404 J/g for Ordinary Portland Cement (OPC; cement type CEM I) of classes 32.5, 42.5, and 52.5.

**Figure 1:**
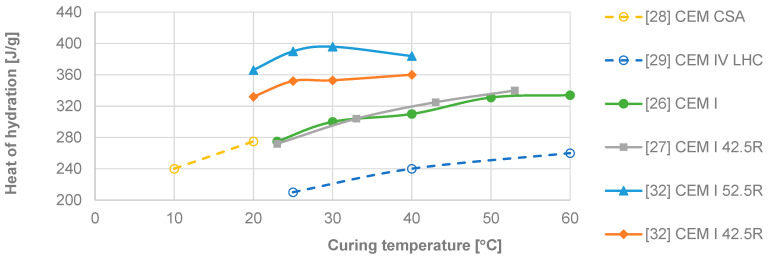
Heat of Hydration of Cement Pastes Under Different Curing Temperatures.

Regarding the variability of heat of hydration results, some values from the literature [21,22,23,24,25,26,27,28,29,30,31] are presented in Table 1. These values refer to cement pastes hydrated in the first 72 h (3 days) and were determined using OPC, except as noted in the last column. The method used was isothermal conduction calorimetry, unless otherwise indicated. Factors influencing the hydration value include the water/cement ratio (W/B) and the curing temperature (T_cur_). Some results from the literature regarding curing temperature are shown in Figure 1.

Even excluding the lower values reported by Pang et al. [29] for low-heat cement (CEM IV), there are discrepancies between the remaining values. Kiernożycki and Błyszko [32] suggest that the heat released by CEM I-type cement pastes increase up to curing temperatures of around 30 °C, after which it decreases. In contrast, other authors did not observe a decrease in heat of hydration up to 60 °C for the same type of cement. For OPC (CEM I) cured at room temperature (20–25 °C), the coefficient of variation is 11%. When considering the W/B parameter, the trendline has an R2 value of 0.564. Despite using the same type of cement and methodology with equivalent curing temperatures, this variability is considerable. Furthermore, Zhang et al. [33] determined that the heat of hydration increased from ~270 J/g (W/B = 0.2) to 393 J/g (W/B = 0.6) in a CEM II cement paste. Thus, high curing temperature and high W/B increase the total heat released, indicating that an increase in temperature in a large mass of concrete due to hydration reactions also increases the heat released. It is also reasonable to assume that the actual temperature in the test piece does not match the curing temperature range.

Xie et al. [34], referencing Chinese (GB/T 200), American (ASTM C150/C150M), European (EN 197-1/EN 14216), and Japanese (JIS R 5210) regulations, state that “The 7-day heat of hydration is required to be between 220 and 270 kJ/kg.” The authors cited in Table 1 also acknowledge that the heat of hydration is a crucial parameter to consider.

**Figure 2:**
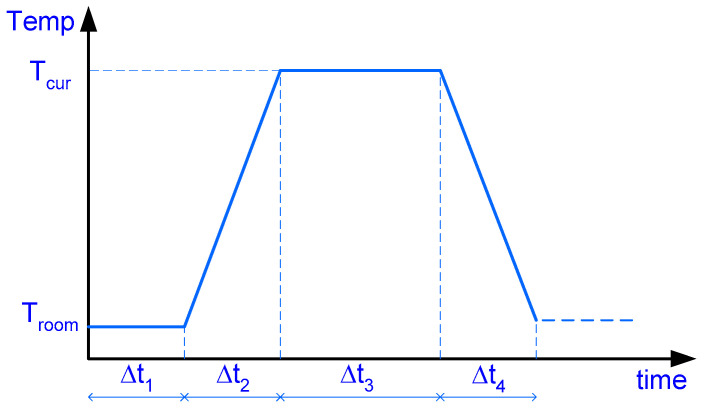
Generic Temperature-Time Diagram for Curing Process.

An important aspect in evaluating the heat of hydration is the curing temperature regime adopted. This process is typically used in factory-produced precast concrete elements to meet the requirement for prestressing tensioning strength. Generally, a regime similar to that shown in Figure 2 is adopted. For typical concretes, the second heat peak occurs between 10 and 13 h, making it unnecessary to consider cases where Δt_1_ ≥ 12 h, which are usually associated with ultra-high-performance concrete [35,36,37]. In these instances, specimens are subjected to high temperatures (90–250 °C) 12 to 72 h after placing the mixture, often after demolding [38].

### 1.2. Curing Temperature on Concrete

For this study, it is relevant to focus on the curing temperature regime applied to concrete specimens immediately after mixing, specifically where Δt_1_ < 3–5 h. In this context, Duan et al. [39] conducted a study with conditions of room temperature ~20 °C, 100 mm cubic samples, a mix composition of 1:3.8:0.32, 1.2% superplasticizer, Portland cement CEM II, unspecified mold, and steam curing. They assumed Δt_1_ = 4 h, with Δt_2_ and Δt_4_ adjusted so that ΔT_i_/Δt = 10 °C/h, and Δt_3_ set at 6, 12, 24, or 48 h, with curing temperatures (T_cur_) of 40 °C, 50 °C, or 60 °C. Their results highlighted that the core temperature of the manufactured sand concrete peaked at about 11–12 h, reaching 43.4 °C, 54.1 °C, and 64.7 °C at curing temperatures of 40 °C, 50 °C, and 60 °C, respectively, consistent with the peak exothermic hydration rate of the cement paste. There was a notable cumulative increase with rising curing temperatures. Secondly, the reduced dimensions of the specimens minimized the additions (cumulative increase). Compared to the standard curing temperature (20 °C), the compressive strength of concrete at 3 days increased significantly (~30%) with higher Δt_3_ and T_cur_. However, at 28 days, a decrease in compressive strength (up to ~9%) was observed for both parameters. Similarly, Zhou et al. [40], citing Vaasudevaa et al. [41], Zhang et al. [42], Gesoglu et al. [43], and Garcia Calvo et al. [44], reported up to a 92% increase in compressive strength on the first day but also noted a 20% decrease at 28 days.

### 1.3. Impact of Curing Temperature on Concrete Strength

Some authors have noted a direct relationship between the heat of hydration and the compressive strength of concrete [27,37,45]. Generally, they argue that high values of heat of hydration correlate with a high degree of overall reaction, which, in turn, correlates with the high strength of the cementitious material. However, there is a discrepancy: it is well-known in concrete technology that high water-to-binder (W/B) ratios significantly deteriorate the compressive strength of concrete [9], yet Zhang et al. [33] demonstrated that the heat of hydration increases substantially with W/B. Zhang et al. concluded that it is the heat of hydration, not temperature, that influences strength.

Wang et al. [46] (room temperature ~20 °C; cement paste with W/B = 0.3; Portland cement CEM I; steam curing; Δt_1_ = 3 h, Δt_2_ and Δt_4_ such that ΔT_i_ / Δt = 15–20 °C/hour, Δt_3_ = 8 h; curing temperatures (T_cur_) of 20 °C, 45 °C, 60 °C, and 80 °C) found that higher curing temperatures resulted in lower compressive strength, up to a 15% reduction. Similarly, Liu et al. [47] (cement mortar mix of 1:0.33:0.45; OPC; 40 mm × 40 mm × 160 mm samples) obtained intriguing results with a two-stage curing temperature regimen: initially at 20 °C, followed by 5 °C. They varied the duration of the first interval: 400 min (6 h 40 min), 1440 min (24 h), or 2000 min (33 h 20 min). The compressive strength at 28 days was ~40 MPa for 400 min of initial curing, but only ~31 MPa for the other two intervals. These results suggest that an initial reduction in curing temperature can enhance the compressive strength of concrete at 28 days.

### 1.4. Hydration Temperature of Concrete

In numerical heat transfer simulations, the heat of hydration is a critical parameter. However, in civil construction practice, the crucial aspect is understanding the impact of the exothermic hydration reaction on the strength characteristics of concrete. This impact is best assessed based on the temperature profile reached by the concrete during the initial hours of hydration. It is important to note that temperature is the only parameter that can be directly measured in any concrete mass; the heat of hydration cannot be directly measured.

Some relevant results from the literature are summarized in Table 2 [45,48,49,50,51,52,53]. Although these values correspond to tests on Ordinary Portland Cement (OPC) (except ref. [49]) conducted under adiabatic or semi-adiabatic conditions, they do not provide conclusive information regarding temperature increases. There are two main limitations: the composition and size of the specimens used. Nevertheless, the variability of the values found is worth noting.

Several authors [52,54,55,56,57] have conducted numerical simulations of heat transfer related to the hydration process. Lee et al. [55], using semi-adiabatic test results of a concrete mix (1:4.6:0.45; 0.7% superplasticizer; CEM IV cement; specific heat not provided), numerically determined a temperature increase of ~38 °C at the center of a large concrete mass (20 m × 15 m × 3 m) at ~140 h. Tahersima and Tikalsky [56], based on the thermal characteristics of a concrete mix (1:6.5:0.49; 0.2% water reducer; blended limestone cement with 25% type C fly ash; heat of hydration ~280 J/g at 72 h and ~320 J/g at 115 h), numerically predicted a temperature rise of ~43 °C in a slab (thickness of 1220 mm) on lean concrete at ~50 h. Shi et al. [57] (room temperature ~20 °C; 400 mm cubic samples; mix composition of 1:4.3:0.29; 1.1% superplasticizer; Portland cement) CEM I (plastic mold; steam curing; Δt_1_ = 2 h, Δt_2_ and Δt_4_ = 2 h, Δt_3_ = 8 h; curing temperature of 60 °C) determined a maximum temperature of 137 °C at 7 h. Using 100 mm cubic molds, the temperature rose to ~71 °C in plastic molds (3 mm thickness) and wood molds (15 mm thickness), or up to ~65 °C in metallic molds (3 mm thickness). These results emphasize the significance of specimen size on temperature profiles.

In addition to the variability of results related to the heat of hydration [20] and the dependence on the test method used [19], it is essential to consider the sensitivity of concrete’s strength properties when cured at temperatures above 60 °C. This sensitivity makes the prediction of strength properties, based on the numerical prediction of the temperature profile during the initial hours of hydration, highly uncertain.

### 1.5. Purpose of the Work and Its Significance

In summary, the mechanical properties of concrete depend on the temperature reached during the first hours of curing. Tensile, compressive, and flexural strengths, along with modulus of elasticity, are commonly used to characterize the strength properties of concrete, with compressive strength being the most critical and typical parameter. The dependence of strength on temperature has not been fully established [37]. The curing temperature at the surface does not necessarily coincide with the core temperature of the concrete specimen [39]. In large concrete masses, the temperature achieved depends on the heat of hydration, which itself depends on the temperature. It is reasonable to accept that the temperature profile during the initial hours of curing is the essential parameter for estimating the strength properties of concrete. Importantly, temperature can be directly measured on-site, whereas the heat of hydration can only be measured indirectly.

The motivation for this work arose from a practical situation involving significantly higher temperatures than those mentioned in Table 2, raising immediate concerns about the material’s strength. While the specific situation is not detailed here due to a lack of data, the key question remains: what is the impact of effective curing temperature during the first hours of cement hydration on the strength properties of concrete? This work, developed over months, attempts to answer this question, acknowledging that the path was often unclear and that much work remains. The results presented here differ somewhat from values found in existing literature and provide an alternative perspective.

In this context, the primary objective of this work is to develop a new methodology capable of performing a truly adiabatic test on a cement mixture within an infinite domain during the initial hours of cement hydration. Given the practical limitations of laboratory conditions (e.g., specimen size, time constraints), adiabatic tests will be conducted on specimens with finite dimensions (~150 to 200 mm). For the test to be considered truly adiabatic, it is essential to ensure that the specimen remains fully insulated from its surroundings throughout the hydration process, meaning no heat is exchanged with the environment. This condition can only be met if the temperature is uniform throughout the specimen, with minimal differences between the temperature at the center and the boundary. Additionally, the cumulative effect of any temperature differences must be reduced to negligible levels. Only by fulfilling these two conditions can the test be considered almost fully adiabatic.

Following the adiabatic test, the second objective is to evaluate whether the cement hydration process has any impact on the strength degradation of the mixture, particularly in terms of compressive strength.

The main significance of this work lies in the development of a new methodology for conducting adiabatic tests. Two key conditions are emphasized in this process: limiting the temperature differences between the interior and boundary of the specimen and accounting for the cumulative effect of these differences. The examples provided serve as applications of this methodology, demonstrating that, when the temperature generated by cement hydration is high, it can indeed lead to a reduction in the concrete’s strength.

In what follows, the description of the study is developed in three main stages. The first stage addresses the challenges of conducting an adiabatic test on cementitious material during the initial hours of hydration. The second stage proposes a method for performing such a test, demonstrating the temperature achievable in a cement mixture. This temperature corresponds to that in a quasi-infinite medium with the same mix. Finally, the third stage presents tests evaluating the impacts of these hydration-induced temperatures on the strength of concrete.

## 2. Materials and Methods

For this study, sand with a relatively rolled shape, collected in Coimbra, Portugal, was used as the fine natural aggregate in the production of mixes. The sand had a particle size fraction of 0/4 mm. The density of the sand particles was 2.64 g/cm³, while the bulk density of the sand itself was 1.6 g/cm³. Based on particle-size analysis, the sand was classified as poorly graded (fines deficit) according to the Unified Soil Classification System, ASTM D 2487-06 [58].

To simulate the curing of the specimens in an adiabatic manner, mimicking an infinite mass, an electric oven was utilized. The oven had free internal dimensions of 58 cm × 98 cm × 80 cm and was capable of reaching temperatures up to 200 °C.

Temperature measurements were taken using type K thermocouples. Compressive strength tests were conducted on 150 mm cube specimens, in accordance with EN 196-1 [59].

The experimental procedure evolved throughout the course of this research, with adjustments made to address specific issues that arose, particularly concerning heat dissipation. The authors found no satisfactory descriptions in the literature regarding the performance of adiabatic tests, which became a significant focus of this work. Therefore, this study not only investigates material properties but also contributes to the development of a novel procedure for conducting adiabatic tests. Many of the specimens tested are not presented here, as they were primarily used for exploratory or improvement purposes.

The preliminary tests, constituting the first stage of the work, were conducted on specimens measuring 150 mm × 150 mm × 100 mm. Table 3 presents the compositions of these specimens, alongside their compressive strength (f_c_), ambient temperature (*T_room_*), and the time range (Δt) required for mortar placement and vibration, starting from the moment water was added to the cement. The cement used in these mixtures was CEM II/A-L 42.5R, manufactured by CIMPOR (www.cimpor.com, accessed on 1 April 2024), which is designed for rapid strength development. This cement consists of 80–94% clinker and 6–20% limestone. It is important to note that the mixtures did not meet the specifications of NP EN 206-1, which requires that strength tests be conducted on 150 mm edge cubes and that compressive strength be measured after 28 days. Therefore, the compressive strength values provided here are indicative only.

A custom mold for specimen NM0912 was constructed using six 20 mm thick Polyvinyl Chloride (PVC) plates in contact with the specimen, surrounded by six 20 mm thick plywood plates. To enhance insulation, 30 mm thick Extruded Polystyrene Foam (XPS) plates were added to create the mold for specimen NM0919. This methodology closely mirrors the processes used in adiabatic or semi-adiabatic tests.

The second stage of the research also included additional mixtures aimed at implementing an adiabatic test: NM1029, NM1102, NM1106 and PM1118. One such mixture, PM1118, incorporated a water reducer (MasterPozzolith 540 plasticizer from BASF, Ludwigshafen, Germany) at a concentration of 1.2% of the cement mass. This additive, recommended by a technician from a ready-mixed concrete company, is appropriate for large volumes of concrete. To minimize the impact of specimen size, a 200 mm cubic mold was constructed from plywood, with its joints sealed using spray-on mastic to prevent leakage during vibration. However, this mold was unsuitable for compression testing due to poor face alignment.

The third stage of testing focused on assessing the effect of the curing temperature profile during the early stages of hydration on concrete strength. A total of 13 steel molds (150 mm cubes) were placed in the oven: 12 cubes for compression testing and 1 cube to monitor the adiabatic process. Additional cubes were cast using the same mixture and cured at ambient temperature in PVC molds. Table 3 lists the four tests conducted in this stage. CEM II/B-L 32.5N cement by CIMPOR, which is suitable for large volumes of concrete and exhibits slow mechanical strength development, was used. According to CIMPOR, this cement is composed of 65–79% clinker and 21–35% limestone.

Due to the large number of cubes required, a vertical-axis concrete mixer (180 dm³) was used. The time intervals (Δt) from the addition of water to the cement until the start of temperature readings were optimized following several training sessions and preparation of the work team. To ensure that the external cubes were cast within the workability window (75 min to initial setting) specified by the cement manufacturer, efficient time management was crucial. A key question that arose was: how much heat is lost during this handling interval?

Test NM0330 served as a control test, conducted with the oven turned off. In the final two tests, crushed limestone aggregate (gravel 1; diameter ~10 mm) was used. For the final test, a superplasticizer (Sika^®^ ViscoCrete^®^-3005 by Sika Portugal, prt.sika.com, accessed on 01 April 2024) was added at a concentration of 1.2% of the cement mass. According to Sika, this superplasticizer is suitable for applications requiring high early strength and self-compacting concrete.

## 3. Preliminary Results of the Experimental Program

**Figure 3:**
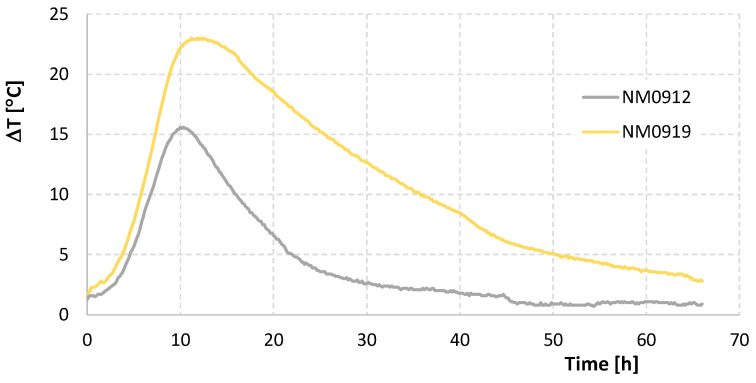
Effective Core Temperature-Time Diagram for Preliminary Tests.

For the first stage of testing, whose specimens are indicated in Table 3, the diagrams showing the effective values of temperature increase measured at the center of the test specimens are presented in Figure 3. Although the mixture ingredients were at room temperature, the initial ΔT of the test was relatively small: 1.3 °C in specimen NM0912 and 1.8 °C in specimen NM0919. The maximum temperature reached by the NM0912 and NM0919 specimens was 15.6 °C and 23 °C, approximately 10 h and 11 h later, respectively. Soon after reaching the maximum value, ΔT began to decrease, more markedly in the less insulated NM0912 specimen and more slowly and prolonged in the better insulated NM0919 specimen. This decrease in temperature occurs because heat losses (proportional to the temperature differential between the specimen surface and the outside) exceed the heat generated by the cement hydration. This aspect significantly complicates the analysis of results for extrapolation to an infinite mass.

This basic procedure has two fundamental criticisms. First, there is always heat loss to the outside (energy dissipation), regardless of the insulation level of the test piece. These losses are highly dependent on the external temperature. Second, the increase in temperature of the mold and insulation requires energy, which is drawn from the cement hydration in the test specimen, and its quantification is not straightforward.

The main conclusion from these two tests is that it is not feasible to perform a fully adiabatic test using this insulation method. Significant heat losses, reflected by the reduction in temperature within the test specimen, indicate that this approach is inadequate for achieving true adiabatic conditions.

## 4. New Methodology for Adiabatic Test

### 4.1. Adiabatic Procedure

To conduct an adiabatic test, simulating an infinite mass, an oven was used to ensure uniform temperature throughout the test piece. Non-uniform temperature in the specimen indicates either dissipation (if the surface temperature is lower) or heat absorption. Thus, the test is adiabatic only if the temperature inside the mold surface matches the temperature within the test piece.

These issues were addressed by placing additional thermocouples: at the center of two faces (face), at two corners (corner), and two at the center (core) of the specimen. Except for those in the center, the thermocouples were placed at the material-mold interface. To determine if heat was being supplied to or removed from the specimen, a methodology was used based on the mold temperature (*T_mold_*) and the sample temperature (*T_sample_* = *T_core_*). The test would be adiabatic if *T_mold_* = *T_core_* at all times. The *T_mold_* temperature was estimated by:

(1) $$T_{mold}=\frac{1}{3}\left(2T_{face}+T_{corner}\right)$$

The difference between *T_mold_* and *T_core_* is defined as Δ*T_ms_*:

(2) $$∆T_{ms}=T_{mold}-T_{core}$$

If Δ*T_ms_* > 0, the specimen receives heat; if Δ*T_ms_* < 0, the specimen gives off heat. Recording this difference over time also allows calculation of the accumulated temporal average value (*AT_ms_*) at a given instant *t*:

(3) $$AT_{ms}\left(t\right)=\frac{1}{t}\int_{0}^{t}∆T_{ms}\left(t\right)dt$$

This does not guarantee a perfectly adiabatic test if *AT_ms_* ~ 0 at the end. The variability of the thermal characteristics of the mix, particularly specific heat, must be considered. Evaporation was controlled in this regard. However, if very low values of Δ*T_ms_* (<1 °C) with alternating signals over short periods are cumulatively maintained, the final test piece temperature would approximately match the *T_core_* temperature minus a value proportional to *AT_ms_*.

Temperature values (*T_core_*, *T_face_*, *T_corner_*, and *T_mold_*) are defined relative to the initial ambient temperature (*T_room_*), representing increases compared to *T_room_*.

**Figure 4:**
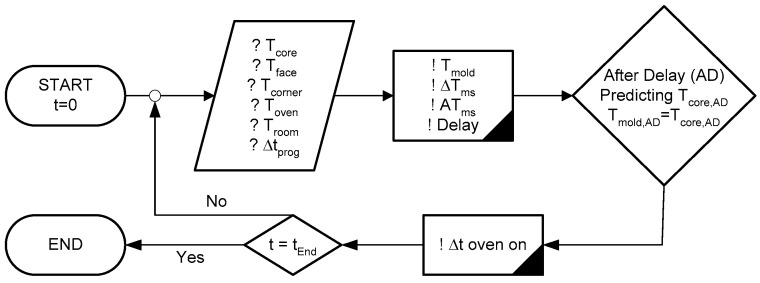
Flowchart of the Adiabatic Process.

Implementing this methodology (as outlined in Figure 4) presents several challenges. First, the temperature variation inside the oven is influenced by factors such as its capacity, internal and external temperatures, and the materials placed inside. Second, achieving the desired mold temperature (*T_mold_*), which accounts for the delay between the oven and the boundary of the mold containing the test specimen, requires careful pre-setting of the oven temperature (T_oven_). Third, predicting the temperature evolution within the mortar demands experience with the process. Several approximation tests were performed. For the first and second steps, it was determined that the delay between turning on the oven and its effect on the specimen boundary was approximately 20 min. To manage this, the oven was programmed in 10-min intervals (Δt_prog_) during the day and 20-min intervals at night.

### 4.2. Adiabatic Tests

In these tests, 10 thermocouples (5 pairs) were used: one pair on two faces, one pair in two corners, two in the center, two inside the oven, and two outside. The final value at each instant was the average of the corresponding thermocouples. If values from thermocouples near the test piece differed significantly, the results were invalidated. Readings were taken every 2 min.

**Figure 5:**
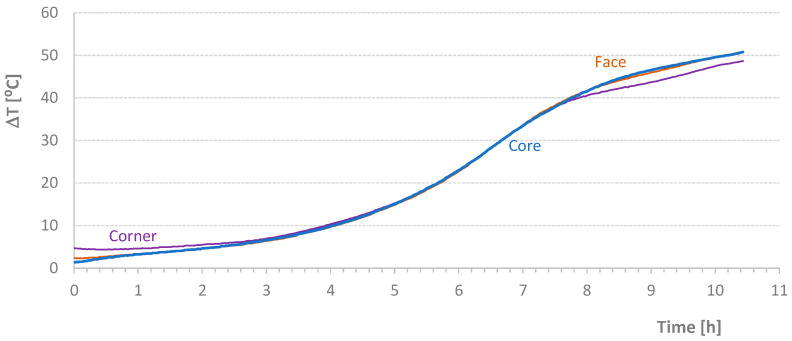
Effective Temperature-Time Diagrams for Test NM1029.

**Figure 6:**
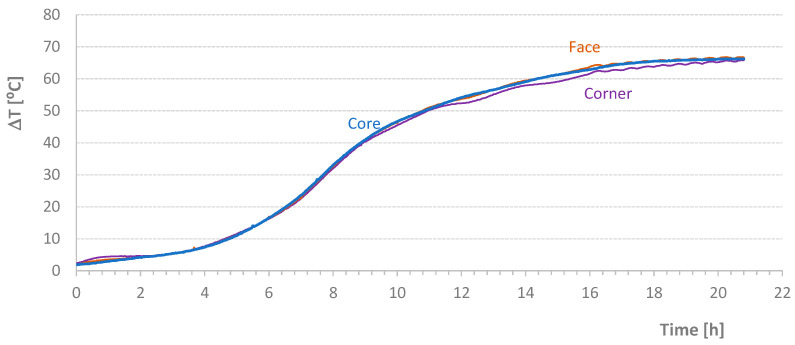
Effective Temperature-Time Diagrams for Test NM1102.

**Figure 7:**
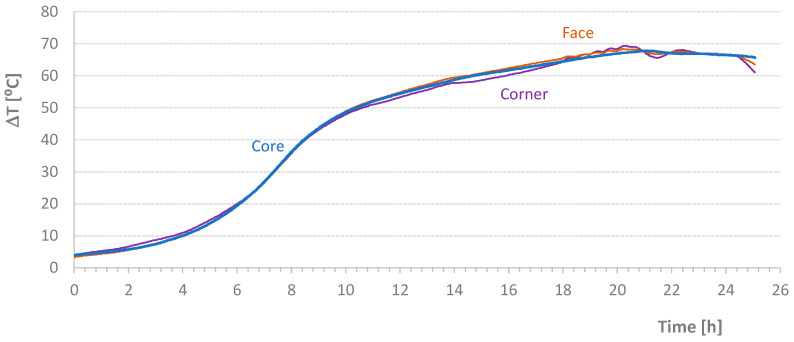
Effective Temperature-Time Diagrams for Test NM1106.

**Figure 8:**
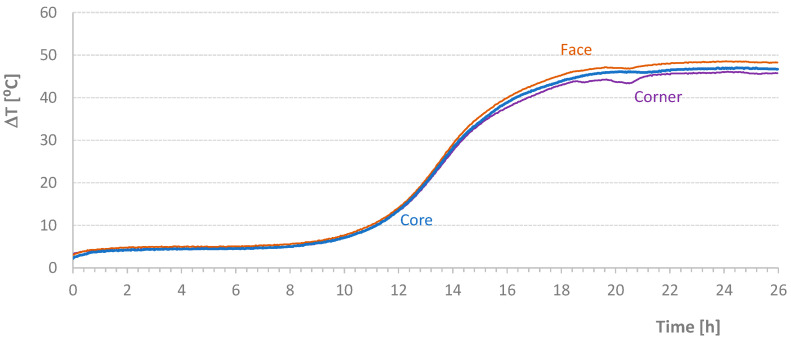
Effective Temperature-Time Diagrams for Test PM1118.

Table 4 shows the average temperature increase over a specified time interval ((ΔT/Δt)_m_) and the effective temperature variation (ΔT) for the mixtures carried out in this second stage of the work. Figure 5, Figure 6, Figure 7 and Figure 8 present graphs of the evolution of core, face, and corner temperatures.

The first test was interrupted due to an imperfection in the programming of the stove resistance controller. Despite this, two important details can be analyzed. First, the values related to the *T_corner_* temperature showed a deviation (visible in the initial graph values), which could potentially invalidate the results. This deviation resulted from preheating the mold beyond the expected temperature of the mixture, 1 °C to 2 °C higher than the initial temperature of the materials, which should coincide with the *T_room_* temperature. Second, the *T_corner_* temperature values were lower at the end of the test when the oven was turned off or when the heat supplied was insufficient to maintain the mold temperature. Initially, this discrepancy was detected at the *T_corner_* temperature, followed by a milder effect at the *T_face_* temperature, as seen at the end of the diagrams in Figure 7.

The temperature velocity (column 2 of Table 4) corresponds roughly to the end of the heat flow acceleration range [34]. It is important to note that the values depend significantly on the width of the range considered for their evaluation, which in this work was approximately 15 min. The second and third values indicate that higher initial temperatures lead to higher temperature speeds. The literature supports this conclusion, stating that the initial temperature of the concrete mix greatly influences the heat generation process [45]. The results also show that higher initial temperatures lead to a final increase in temperature, although the increase is relatively small.

Notably, in the last test where a plasticizer was used, the maximum accumulated temperature was reduced by approximately 19.3 °C, almost 30%, which is quite significant. This result is not found in the literature. Li et al. [3] report a decrease of up to 5–8 °C in the maximum concrete temperature when using plasticizers.

**Figure 9:**
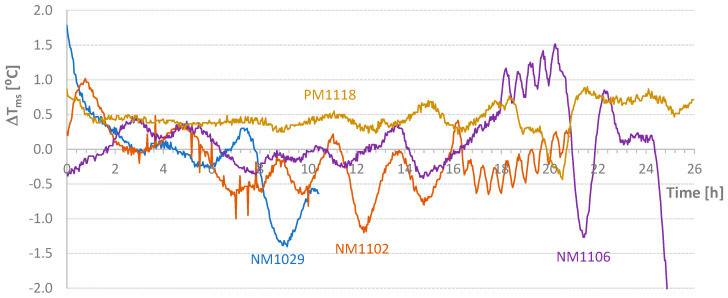
Diagrams of Temperature Difference Between Mold and Samples.

**Figure 10:**
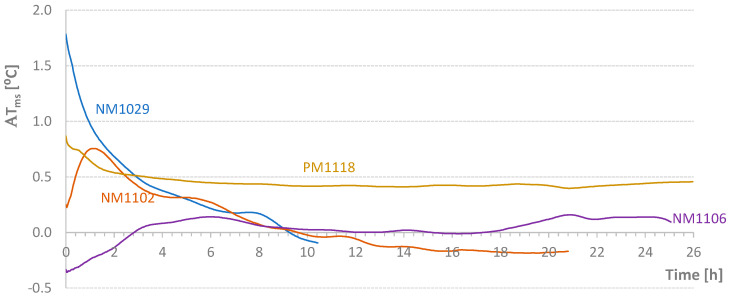
Temporal Average Value of the Temperature Difference Between Mold and Samples.

**Figure 11:**
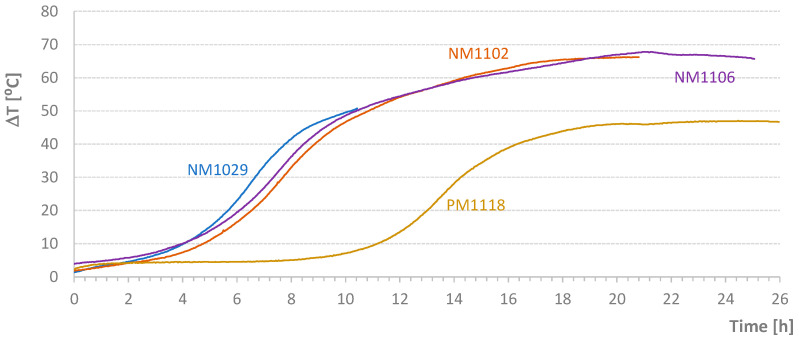
Diagrams of Temperature Increases in Adiabatically Cured Specimens.

For the tests indicated in Table 4, the values relative to the parameter Δ*T_ms_* defined in Equation (2) are presented in Figure 9. Except for small periods of time, it was possible to maintain the temperature difference within the range of [−1 °C; 1 °C]. However, the diagrams require critical analysis, particularly the final part of the NM1106 test. Figure 9 also shows the initial addition of heat to the NM1102 test. Conversely, at the beginning of the process, some heat was removed from the NM1106 specimen. Figure 10 displays the *AT_ms_* values defined in Equation (3). Except for the PM1118 case (*AT_ms_* = 0.46 °C), the temporal average value of the temperature difference between mold and samples was about 0.2 °C or less. This approximation would be excellent if the heat of hydration did not depend on temperature at each instant. Nevertheless, this result is still classified as very good. In Figure 11, graphs of the final values of the accumulated effective temperatures (*T_core_*) are presented. It is important to highlight the agreement of the values of the first three tests and the significant reduction in temperature evolution in the fourth test (PM1118). The initial higher values for the NM1029 test likely result from the initial *T_room_* and *T_mold_* temperatures. Regarding the PM1118 diagram (which incorporated a plasticizer), it is also important to note, in addition to the lowest temperature reached, that the temperature increase phase occurs approximately 7 h later than in the other tests. These two aspects combined in a concrete mass, regardless of its heat dissipation capacity, can lead to significantly lower temperature increases.

## 5. Temperature-Impact on Strength

The third step of this work involves evaluating the impact of the curing temperature profile during the first hours of hydration on the strength of the concrete. Table 5 lists the four tests performed and the temperature increase (ΔT) relative to the ambient temperature (*T_room_*).

### 5.1. Adiabatic Curing

Several difficulties were encountered during this phase of the work (excluding numerous canceled tests). First, controlling the oven with three shelves proved challenging. During heating periods, the temperature oscillations on the upper shelf were of lower amplitude compared to the middle shelf, which in turn had lower amplitudes than the lower shelf. The cycle amplitude on the lower shelf (up to 3 °C) depended on the heating period relative to the control cycle (20 to 25 min at night). Although these oscillations were not detectable inside the specimen and therefore did not affect the adiabatic curing objective, they highlight the challenge of maintaining uniform temperature control. The second issue pertained to the specimen size. It was found that controlling the adiabatic test is substantially more difficult with 150 mm cubic specimens.

**Figure 12:**
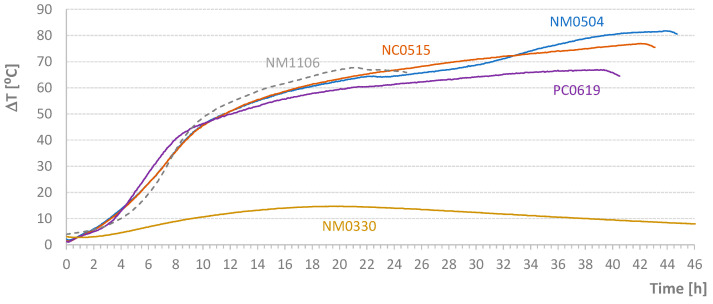
Diagrams of the Temperature Increases of the Third Test Specimens.

Figure 12 shows the diagrams relating to the temperature increases (ΔT) of the adiabatically cured specimens. For comparison, the previous test NM1106 (indicated in dashed line) is included in this figure. The ΔT achieved in test NM0330 (control) is similar to the value achieved in the first preliminary test NM0912, particularly at *t* = 19 h. This similarity might suggest that the type of cement and additives play a crucial role in defining the heat release acceleration range. However, upon examining the other curves, notably in comparison with the NM0912 diagram, this conclusion does not hold. In semi-adiabatic tests, it is difficult to draw definitive conclusions about the peaks of heat released due to two primary reasons: firstly, the heat of hydration at each instant is dependent on the temperature itself, and secondly, if the temperature field is not uniform, the process inevitably becomes random in nature.

**Figure 13:**
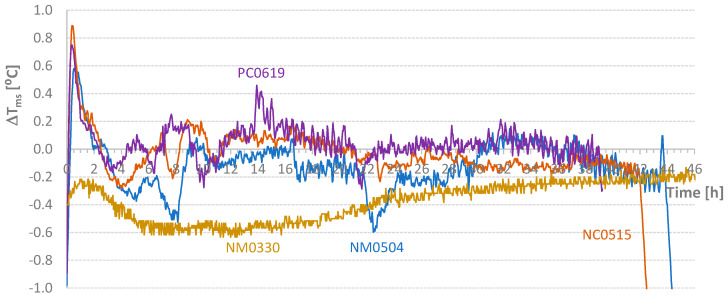
Diagrams of Temperature Differences of the Third Test Specimens.

**Figure 14:**
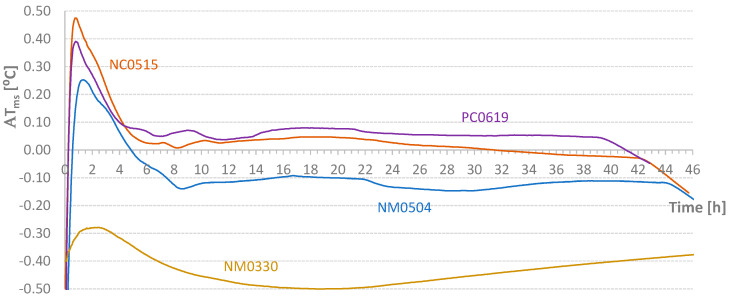
Temporal Average Value of the Temperature Difference of Third Samples.

Figure 13 and Figure 14 present the diagrams corresponding to the temperature differences between the test piece and the mold (Δ*T_ms_*) and the temporal average of accumulated values (*AT_ms_*; see Equation (3)). The control test (NM0330) provides insight into the boundaries of an adiabatic test. In this case, it is noteworthy that the cubes inside the oven were not cured at room temperature. The bottom shelf temperature reached a maximum of 28 °C, while the top shelf reached 31 °C, and the specimen itself reached 31.5 °C. The maximum temperature difference for the mold was 0.6 °C. This value, along with the average accumulated value (*AT_ms_* ~ 0.5 °C), serves as a reference for this semi-adiabatic test. The key question is the acceptable limits for a test to be considered adiabatic. Based on the authors’ experience, Δ*T_ms_* differences must be within the range of −0.5 °C to 0.5 °C, with alternating signs to ensure that the accumulated average value (*AT_ms_*) at the maximum temperature point is less than 0.1 °C. For instance, test NM0504 does not meet these limits. As shown in the figures, this sample was almost always in a temperature deficit, although it reached the highest temperature.

### 5.2. Compressive Strength Tests

**Figure 15:**
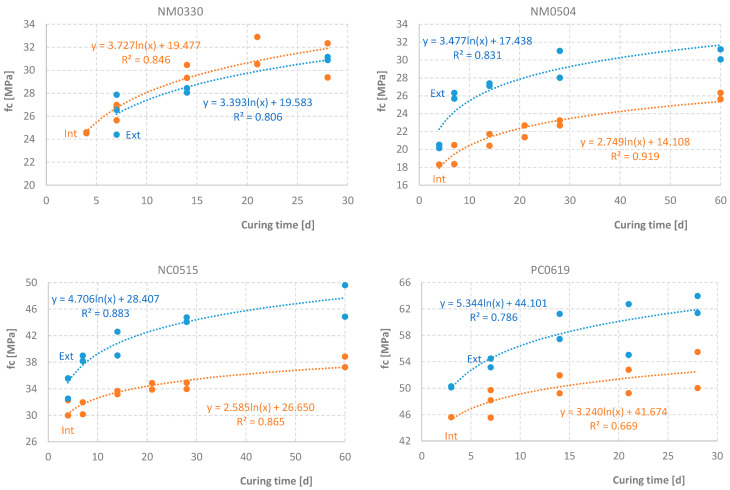
Compressive Strength of Third Samples.

Regarding compressive strength (fc), cubes on different days were tested—days 4, 7, 14, 21, and 28. Subsequently, using a methodology proposed by Neville [9], a logarithmic trendline to define the expected strength values (f_c,e_) was established. Figure 15 shows the determined values for the exterior cubes (Ext; cured at room temperature) and the cubes cured inside the oven (Int), along with the corresponding trendlines and equations. Table 6 shows the expected compressive strength values on day 7 and day 28 and the corresponding deviations, calculated as the quotient of the difference between values relative to the reference value.

Using the cubes cured at room temperature as a reference, it was found that cubes cured in a semi-adiabatic process, up to a temperature of 14.7 °C above the *T_room_* temperature (16.9 °C), exhibited a negligible increase in compressive strength of 3.3% at 28 days. In contrast, the reduction in compressive strength for mortar cured under adiabatic conditions (maximum temperature *T_max_* = 101.7 °C) was significant at 20% after 28 days. A similar reduction was observed for concrete without additives (*T_max_* = 96.9 °C). However, when an accelerating superplasticizer was added to the concrete (*T_max_* = 92.1 °C), the reduction in compressive strength was only 15%. Notably, the trend of these deviations increased from day 7 to day 28.

## 6. Conclusions and Future Thoughts

A new methodology for conducting an adiabatic test of a cement mixture was proposed in this work. The primary objective was to characterize the temperature evolution of a cementitious mixture in an infinite medium during the first hours of hydration. The secondary objective was to evaluate the impact of this temperature profile on the material’s strength. The following conclusions can be drawn from this study:

- The type of cement and the additives used are crucial in defining the heat release acceleration range and the maximum temperature reached by an infinite mass.
- In a typical concrete mixture (1:2.5:0.4) without additives, temperatures can rise to around 80 °C above the ambient temperature.
- Adding a setting acceleration additive to the mixture reduces the temperature increase by approximately 12 °C, while a retardation additive decreases the temperature by about 19 °C.
- For normal, adiabatically cured cements, the second heat peak typically occurs between 10 and 13 h. However, there are situations where the second heat peak may not occur within this interval. The release of hydration heat depends on the mixture’s temperature at each moment.
- In typical concrete mixtures, the temperature increase in an infinite mass can reduce compressive strength by up to 20%.

### Future Work

The following ideas for future research are proposed:

- The thermal characteristic that most significantly influences the temperature profile during the first hours of cement hydration is, without a doubt, the heat of hydration. As noted, this parameter also depends on temperature. Therefore, accurately assessing the heat of hydration of cement can only be achieved through an adiabatic test. The accuracy of other parameters can be more easily approximated since these parameters do not exhibit significant variability concerning the temperature range. Their values essentially depend on the mixture’s composition, porosity, and humidity degree.
- A relevant question concerns the use of results from semi-adiabatic tests or supposed adiabatic tests. Since the heat of hydration of cement is temperature-dependent, the experimentally determined value would only be correct if the hydration process of cement were exposed to the same temperature profile.
- Another pertinent question is related to the evaluation methods of the heat of hydration of concrete, such as the Lagavant method or isothermal conduction calorimetry method mentioned in the introduction. Moreover, the variability highlighted, the value relating to the first hours of hydration depends on the process temperature. The temperature profile of the cement paste used to evaluate the heat of hydration does not coincide (at least theoretically) with that of the concrete mix, as part of the heat generated is used to heat the aggregates (whose thermal characteristics are also important). Therefore, it is questionable to assume that the value obtained for the heat of hydration in a cement paste applies to concrete in numerical simulations.
- An excellent avenue for investigation would be controlling the temperatures of cementitious mixtures using setting retardant additives.

## Figures and Tables

**Table 1:** Heat of Hydration of Cement Pastes.

Ref.	W/B	T_cur_ [°C]	f_c_ [MPa]	q [J/g]	Obs.
[21]	1:0.5	20	~26	~260	
		50	--	~380	
[22]	1:0.24	Ambient	--	212	
[23]	1:0.45	Ambient	~23	~290	
[24]	1:0.4	20	~66	222	
[25]	1:0.3	20	~58	250	
[26]	1:0.35	23	--	~260	
	1:0.45	23		~273	
	1:0.55	23		~300	
	1:0.45	60		~334	
[27]	1:0.4	23	~60 @ 14 d	272	Mortar with 1:2.6:0.4 composition
		53	~42 @ 14 d	340	
[28]	1:0.5	10		~240	CSA
		20		~275	
[29]	1:0.38	25		210	LHC
		40		240	
		60		260	
[30]	1:0.5	20	~28	175	LCM
[31]	1:0.5	25		~305	FPC; THFDC

Ref. means bibliographic reference; W/B means water to binder proportion; T_cur_ means curing temperature; f_c_ means compressive strength at day 28; q means heat of cement hydration at 72 h (3 d); Obs. means additional observations; “~” means an approximate value taken from a graph; “@nd” means at n day; CSA means Calcium Sulfoaluminate cement; LHC means low-heat Cement; LCM means lime-based composite cementitious material; FPC means Ferriby Portland cement (11% of C4AF); THFDC means Tonical Heat Flow Differential Calorimeter.

**Table 2:** Temperature Increase of Cement Mixes.

Ref.	Composition	Additive	f_c_ [MPa]	ΔT [°C]	Obs.
[48]	1:5.42:0.49	--	~35	~46 @ 250 h	q ~ 300 J/g @ 120 h when T_cur_ = 23 °C or 33 °C or 43 °C
[49]	1:4.1:0.37	2%	~69	~36 @ 80 h	blast furnace cement (Cem III) and 300 mm cubes isolated with 30 mm thick thermal foam sheets
[50]	1:6.82:0.4	0.7%	47.4	~40 @ 28 d	q ~ 229 J/g @ 3 d; 80 dm^3^ sample in adiabatic condition
	1:8.92:0.5	0.9%	~37	~35 @ 28 d	
[45]	1:3.14:0.28	1.8%	~57 @ 7 d	52.7 @ 3 d	q = 259, 283 and 285 J/g @ 3 d, respectively; special calorimeter apparatus and cylindrical test samples (ϕ = 240 mm; h = 300 mm)
	1:4.11:0.43	--	--	51.0 @ 3 d	
	1:6.58:0.53	--	--	35.4 @ 3 d	
[51]	1:6.40:0.55	--	32.8	11.5 @ ~15 h	cylindrical test samples (ϕ = 150 mm; h = 300 mm) in a plywood box isolated with 76 mm of thick polystyrene
[52]	1:3.7:0.3	0.6%	77.2	~37 @ 40 h	q ~ 230 J/g @ 3 d; q ~ 280 J/g @ 10d; SCC; 2 m cubic block
[53]	1:5.2:0.5	--	~46	~30 @ 18 h	0.5 m cubic concrete block inside a plywood mold lined with polystyrene insulation (thickness of 16 mm)

Composition is the cement: aggregates: water proportions; Additive means the use of superplasticizer (% of cement); f_c_ means the mean value of compressive strength at 28 days; ΔT means the adiabatic temperature rise; “~” means an approximate value taken from a graph; “@nh” means “at n hour”; “@nd” means “at n day”; q means the specific heat of cement hydration in concrete; T_cur_ means curing temperature; SCC means self-compacting concrete.

**Table 3:** Mixes Compositions.

Test	Composition	f_c_ [MPa]	T_room_ [°C]	Δt [min]
NM0912	1:2.5:0.53	37.8 @ 19 d	24.2	10
NM0919	1:2.5:0.55	34.5 @ 32 d	23.1	10
NM1029	1:2.5:0.53	--	23.5	16
NM1102	1:2.5:0.53	--	20.5	14
NM1106	1:2.5:0.53	--	18.1	13
PM1118	1:2.5:0.45	--	18.2	14
NM0330	1:2.5:0.5	--	16.9	40
NM0504	1:2.5:0.47	--	19.9	25
NC0515	1:2.73:0.38	--	20.0	30
PC0619	1:2.96:0.29	--	25.2	30

“NM” means normal mortar; “PM” means plasticized mortar; “NC” means normal concrete; “PC” means plasticized concrete; “NMmmdd” adds the month and day date to NM test; mix composition in cement: aggregates: water proportions by weight.

**Table 4:** Second Stage Mixes Compositions.

Test	(ΔT/Δt)_m_ [°C/h]	ΔT [°C]
NM1029	10.9 @ (6.4 h–6.7 h)	Not evaluated
NM1102	9.7 @ (7.7 h–8.0 h)	66.2 @ 75 h
NM1106	9.6 @ (7.4 h–7.7 h)	65.8 @ 90 h
PM1118	8.4 @ (13.2 h–13.5 h)	46.7 @ 90 h

**Table 5:** Third Stage Mixes Compositions.

Test	Composition	ΔT [°C]
NM0330	1:2.5:0.5	14.7 @ 19 h
NM0504	1:2.5:0.47	81.8 @ 44 h
NC0515	1:2.73:0.38	76.9 @ 42.1 h
PC0619	1:2.96:0.29	66.9 @ 38.9 h

**Table 6:** Expected Deviations of Compressive Strength.

Test	NM0330	NM0504	NC0515	PC0619
Ext; f_c,e_ @ 7 d [MPa]	26.2	24.2	37.6	54.5
Int; f_c,e_ @ 7 d [MPa]	26.7	19.5	31.7	48.0
Δf_c,e_ @ 7 d	2.1%	−20%	−16%	−12%
Ext; f_c,e_ @ 28 d [MPa]	30.9	29.0	44.1	61.9
Int; f_c,e_ @ 28 d [MPa]	31.9	23.3	35.3	52.5
Δf_c,e_ @ 28 d	3.3%	−20%	−20%	−15%

“Ext” means mix cured at room temperature; “Int” means samples cured under adiabatic conditions; “f_c,e_” means expected value of compressive strength; “Δf_c,e_” means deviation of Int expected value relative to Ext.

## Data Availability

The original contributions presented in the study are included in the article, further inquiries can be directed to the corresponding author.
